# Estimation of the number of women of reproductive age in need of preventive chemotherapy for soil-transmitted helminth infections

**DOI:** 10.1371/journal.pntd.0006269

**Published:** 2018-02-12

**Authors:** Denise Mupfasoni, Alexei Mikhailov, Pamela Mbabazi, Jonathan King, Theresa W. Gyorkos, Antonio Montresor

**Affiliations:** 1 Department of Control of Neglected Tropical Diseases, World Health Organization, Geneva, Switzerland; 2 Department of Epidemiology, Biostatistics and Occupational Health, McGill University, Montréal, Québec, Canada; 3 WHO Collaborating Centre for Research and Training in Parasite Epidemiology and Control, McGill University, Montréal, Québec, Canada; Swiss Tropical and Public Health Institute, SWITZERLAND

## Abstract

**Background:**

Soil-transmitted helminth infections are among the most common infections in developing countries. Globally, as many as 2 billion people are considered to be at risk for soil-transmitted-helminth (STH) infections. Preschool children (PSAC), school-age children (SAC) and women of reproductive age (WRA) are at high risk of STH-attributable morbidity and preventive chemotherapy (PC) for STH is recommended by the World health Organization (WHO).

**Methodology/Principal findings:**

Over the last five years, PC coverage in PSAC and SAC has gradually increased, while coverage in WRA has lagged. Estimating the numbers of WRA in each endemic country would inform scale-up in this group. A two-step process was used: 1) total numbers of girls and women between 15 and 49 years of age were obtained from the United Nations World Population Prospects 2015 database; and 2) the proportion in need of PC was obtained primarily from extrapolation from the WHO PC Databank. WRA were divided into four sub-groups reflecting different reproductive life stages, each having a potentially different interface with the health care system and, consequently, presenting different opportunities for intervention strategies.

Worldwide, we estimated that 688 million WRA in 102 countries were in need of PC for STH in 2015. The South-East Asia (49%) and Africa regions (26%) had the highest numbers. Adolescent girls accounted for 16%, while pregnant and lactating women each represented 10%. Over 25 million pregnant women alone were estimated living in areas where the prevalence of hookworm and *T*. *trichiura* infection was ≥ 20%. Approximately 20% of at-risk WRA had received deworming with albendazole through the Global Programme to Eliminate Filariasis.

**Conclusions/Significance:**

To close current gaps in coverage, numbers of WRA in need of PC for STH are essential for operational strategies to control STH infection.

## Author summary

Soil-transmitted helminths (STH) are intestinal worms that cause morbidity in many people worldwide; children and women of reproductive-age (WRA) are the group most at risk of these infections. The primary control strategy is the periodic anthelminthic treatment of people at risk of STH. Over the last five years, the numbers of children receiving deworming have increased but coverage in WRA has remained low. We compiled data on the number of WRA in STH-endemic countries and estimated the number of WRA in need of PC. This will enable us to develop the necessary operational strategies to control STH infection in WRA.

## Introduction

Soil-transmitted helminth (STH) are infections caused by the nematodes *Ascaris lumbricoides* (roundworm), *Ancylostoma duodenale* and *Necator americanus* (hookworms), and *Trichuris trichiura* (whipworm). Humans acquire these infections from soil contaminated by faeces of STH-infected individuals.

Approximately 2 billion people worldwide are considered to be at risk of STH infections [1]. The primary strategy recommended by the World Health Organization (WHO) to control STH is preventive chemotherapy (PC): the periodic administration of anthelminthic medicines to population groups that are particularly at risk of the morbidity caused by STH [2]. PC should ideally be provided in combination with other measures like health education, improved sanitation and waste management.

The population groups at high risk for STH morbidity include preschool children (pre-SAC) (1–4 years), school-age children (SAC) (5–14 years) and women of reproductive age (WRA) (15–49 years) [3]. STH causes significant nutritional morbidity [4]. It has been estimated that the total number of disability-adjusted life years (DALYs) lost due to STH infections is: between 1.2 and 10.5 million for *A*. *lumbricoides*; between 1.8 and 22.2 million for *T*. *trichiura*; and between 1.6 and 6.4 million for hookworm [5, 6]. The wide ranging estimates result from the variability in extrapolating published prevalence and intensity data obtained from localized surveys conducted in endemic areas over time.

The total number of DALYs lost by WRA is estimated at 680 000 [7].

Evidence suggests that WRA, and especially pregnant women, are at increased risk of iron deficiency anaemia due, in part, to whipworm and hookworm infections [8, 9]. In 2011, 500 million non-pregnant women and 32 million pregnant women aged 15–49 years were estimated to be anaemic with half due to iron deficiency [10]. Anaemia and iron deficiency anaemia impair the health and well-being of women and increase the risk of adverse maternal and neonatal outcomes. A systematic review of hookworm-related anaemia in pregnant women found a consistent relationship between heavy intensities of hookworm infection and lower levels of haemoglobin [6]. The WHO global nutrition target is to reduce maternal anaemia by 50% by 2025 [10] and one of the recommended control strategies is preventive chemotherapy (PC) to reduce STH in WRA, especially in areas where hookworm infection predominates [11, 12].

Despite WRA being among the groups at highest risk for STH-attributable morbidity, actions to increase PC coverage in WRA have been limited. Few countries implement deworming programmes targeting WRA and fewer still record or report data in any systematic manner. One noted exception has been the community-based programmes for the elimination of lymphatic filariasis which include administration of the deworming drug albendazole to WRA within the household.

We consider that one of the possible reasons for the low coverage of PC in WRA is the fact that, in reality, WRA is a heterogeneous group and only a small proportion might have access to a deworming programme. WRA can be divided into four sub-groups representing distinct phases of a woman’s reproductive lifespan, each with its different interface with the health care system: adolescent girls from 15 to 19 years of age are not usually included in school deworming; pregnant and lactating women may be more easily reachable through health services and facilities; and non-pregnant and non-lactating women may only be reachable through community-based programmes. Additional benefits and challenges associated with deworming programmes for each sub-group have recently been summarized [13].

The purpose of this article is to establish, as accurately as possible, the numbers of WRA who are in need of PC for STH in each of the four WRA sub-groups. An appreciation of the numbers of WRA treated within the Global Programme to Eliminate Lymphatic Filariasis (GPELF) will be taken into account and the coverage gap will be identified. The numbers provided here will be the basis for the development of appropriate strategies to reduce STH- attributable burden of disease in WRA.

## Methods

**Fig 1:**
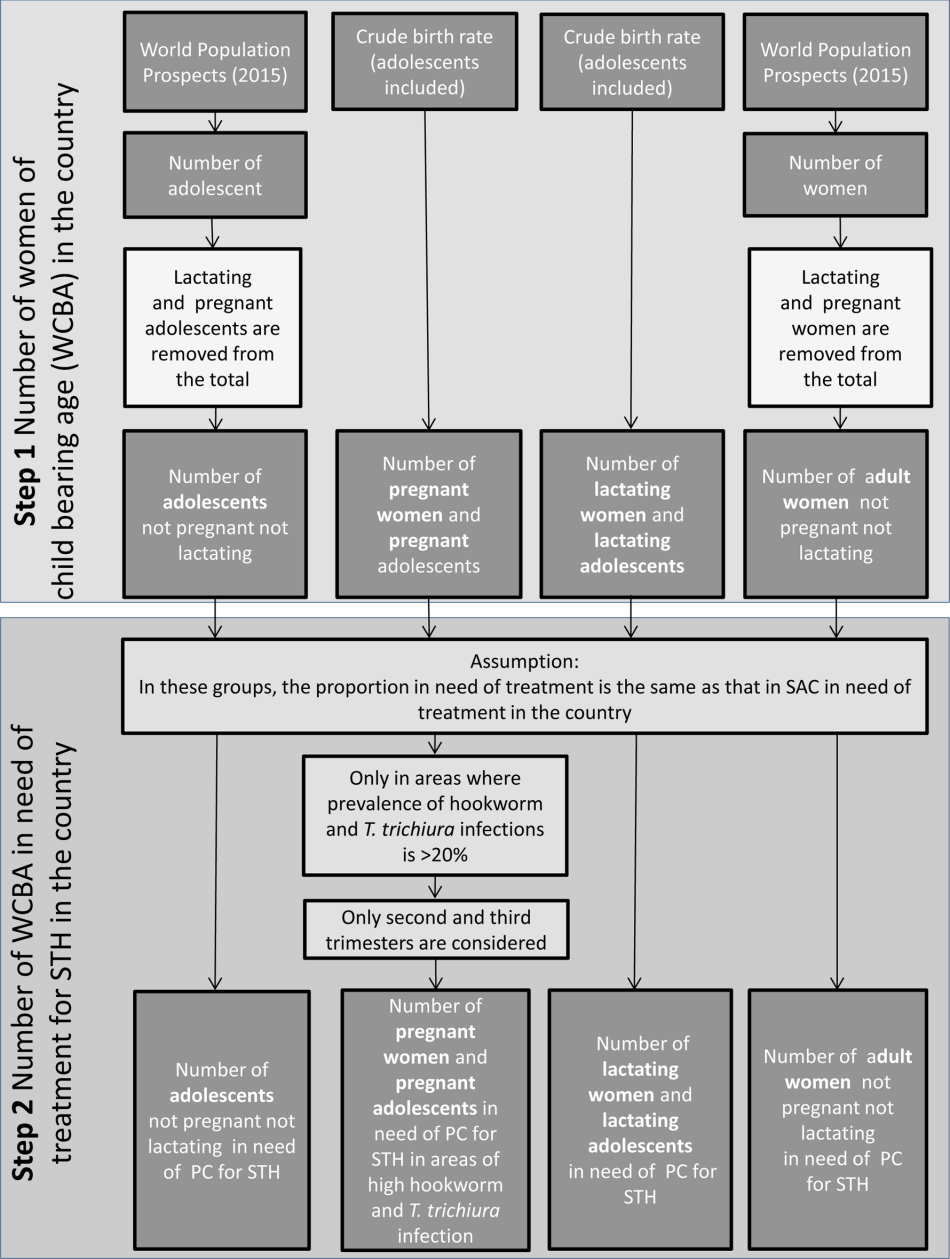
Visualization of the two-step process used to estimate the number of women of reproductive age in need of preventive chemotherapy for soil-transmitted helminth infections in each endemic country.

To estimate the number of WRA (overall and for each sub-group) in need of PC in each STH-endemic country, a two-step process was followed: first, numbers of all girls and women between 15 and 49 years of age were obtained from the United Nation World Population Prospects 2015, a reliable and publicly accessible data source; and second, the proportion in need of PC was obtained from extrapolation from available PC databases maintained by the World Health Organization. The process is described in the following paragraph and summarized in Fig 1.

### Calculating the number of women of reproductive age in STH-endemic countries

The World Population Prospects 2015 Revision database, published by the United Nations Population Division, reports the population for each country by sex and age group (in intervals of 5 years) [14]. From this source, we obtained the population numbers corresponding to WRA (i.e. 15–19; 20–24; 25–29; 30–34; 35–39; 40–44; and 45–49 years of age) for each of the 102 countries considered endemic for STH by WHO [15].

We then assembled these numbers into the following four WRA sub-groups:

1. Adolescent girls not pregnant and not lactating (15 to 19 years of age)
2. Pregnant women (15 to 49 years of age)
3. Lactating women (15 to 49 years of age)
4. Adult women not pregnant and not lactating (20–49 years of age)

Of note we recognize that these categories may not be strictly mutually exclusive since a small number of lactating women could also be pregnant.

To estimate the number of adolescent girls, we extracted the numbers of females 15–19 years of age as reported in the World Population Prospects [14] and excluded the number of pregnant and lactating adolescents because they would be included in the categories of pregnant and lactating women. The number of pregnant adolescents was estimated by applying the adolescent fertility rate in each country [14] to the number of adolescent girls. We assumed that all pregnant adolescents were lactating for 12 months.

The number of pregnant women was assumed to be equal to each country's crude birth rate [14]. This number also includes pregnant adolescents. We recognize that this number is an underestimation, given that any pregnancy not resulting in a livebirth would not be included in this number.

Since the majority of lactating women breastfeed for one year [16], we consider that all pregnant women breastfeed for 12 months after delivery.

The number of adult women not pregnant and not lactating was obtained by subtracting the number of adult pregnant and lactating women from the total number of adult women of reproductive age.

### Estimating the number of women of reproductive age in need of PC for STH

To estimate the number of WRA in need of PC for STH in each country we assumed that, if school-age children (SAC) had previously been determined to be at risk of STH-attributable morbidity in a country, then WRA would be similarly at risk.

We recognize that STHs have species-specific age-prevalence and age-intensity curves [17] and that there are different species-specific prevalence and intensity peaks. Therefore, the occurrence of the different STH species in WRA is not exactly the same as what would be observed in SAC. Nonetheless, it is reasonable to consider that the STH prevalence levels in SAC are a strong indicator of environmental contamination and, consequently, of the potential exposure to STH infection which would be experienced by WRA. In addition, WHO collects data on STH prevalence in SAC from all countries and these data are continuously updated by countries when surveys are conducted (for example, the recent mapping exercises conducted by the WHO AFRICA region [18]). By using this large amount of data already available for SAC in most STH-endemic countries (collected over many years), it is possible to immediately identify areas in need of control programmes for WRA. For this reason, we used the proportion of SAC in need of PC for STH calculated from the WHO PCT databank [15] as the proportion for WRA in need of PC for STH in our estimates (e.g. in a country where 50% of SAC are in need of treatment, 50% of adolescent girls would also be considered in need of PC for STH).

The number of adolescent girls in need of PC for STH in each STH-endemic country was estimated by applying the same proportion as needed for SAC in the country.

WHO recommends PC for STH in pregnant women only in areas where hookworm and *T*. *trichiura* infections have a prevalence of 20% or more [19, 20]. To estimate this number, first, for each country, the same proportion as that of SAC in need of PC for STH was applied to the population of pregnant women;

To calculate the number of pregnant women living in areas where hookworm and *T*. *trichiura* prevalence is 20% or greater, we used sub-national data available in WHO or the data on species from Pullan *et al* [6] and only countries with hookworm and *T*.*trichiura* prevalence equal or greater than 20% were considered in this case.

The number of lactating women in need of PC for STH in each STH-endemic country was estimated by applying the same proportion as that of SAC in need of PC for STH to the total estimated number of lactating women.

The number of adult women not pregnant and not lactating in need of PC for STH was estimated by applying the same proportion as that of SAC in need of PC for STH to the total estimated number of adult women in each STH-endemic country minus the number of adult pregnant and lactating women.

**Fig 2:**
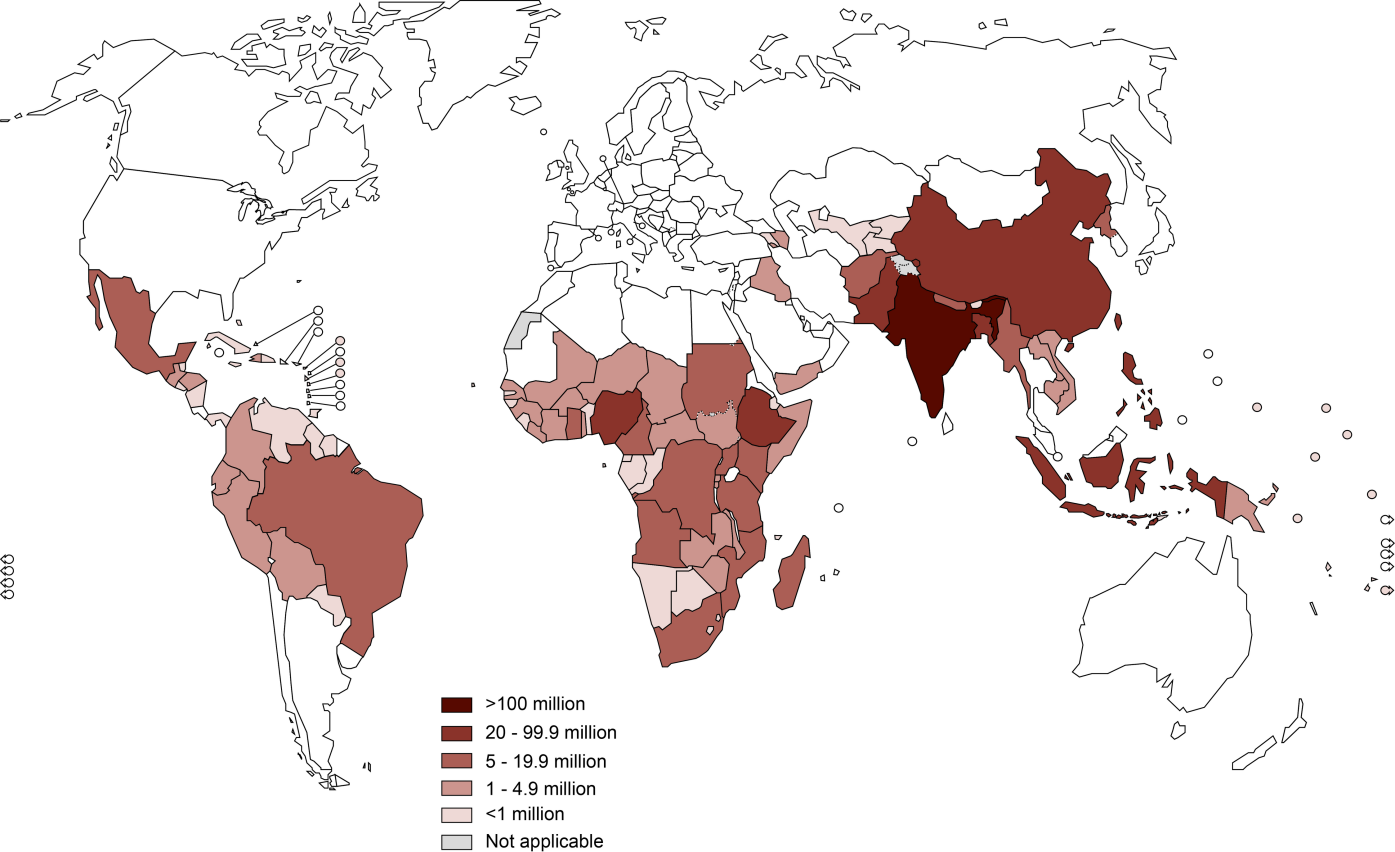
World map showing the estimated number of women of reproductive age in need of preventive chemotherapy for STH, by country, in 2015.

With the estimated number of WRA in need of PC for STH, a map was generated (Fig 2).

### Estimating the number of WRA treated for STH through the Global Programme to Eliminate Lymphatic Filariasis (GPELF)

Those lymphatic filariasis (LF)-endemic countries that have implemented Mass Drug Administration (MDA) to eliminate lymphatic filariasis report the total number of people treated to WHO on an annual basis. Most of the countries collect and send data that are not disaggregated by age and sex. Studies on coverage and compliance with LF MDA show that differences in treatment coverage between males and females, and between age groups is often setting- specific [21, 22].To simplify the estimation of the number of WRA treated through GPELF in 2015, we assumed that the MDA was provided at the same level in all eligible population groups (e.g. if, in a country, MDA covered 70% of the population, it was assumed that 70% of eligible WRA would also have been covered). Pregnant women are excluded from LF MDA and therefore would not be treated.

## Results

### Number of women of reproductive age in STH-endemic countries

**Fig 3:**
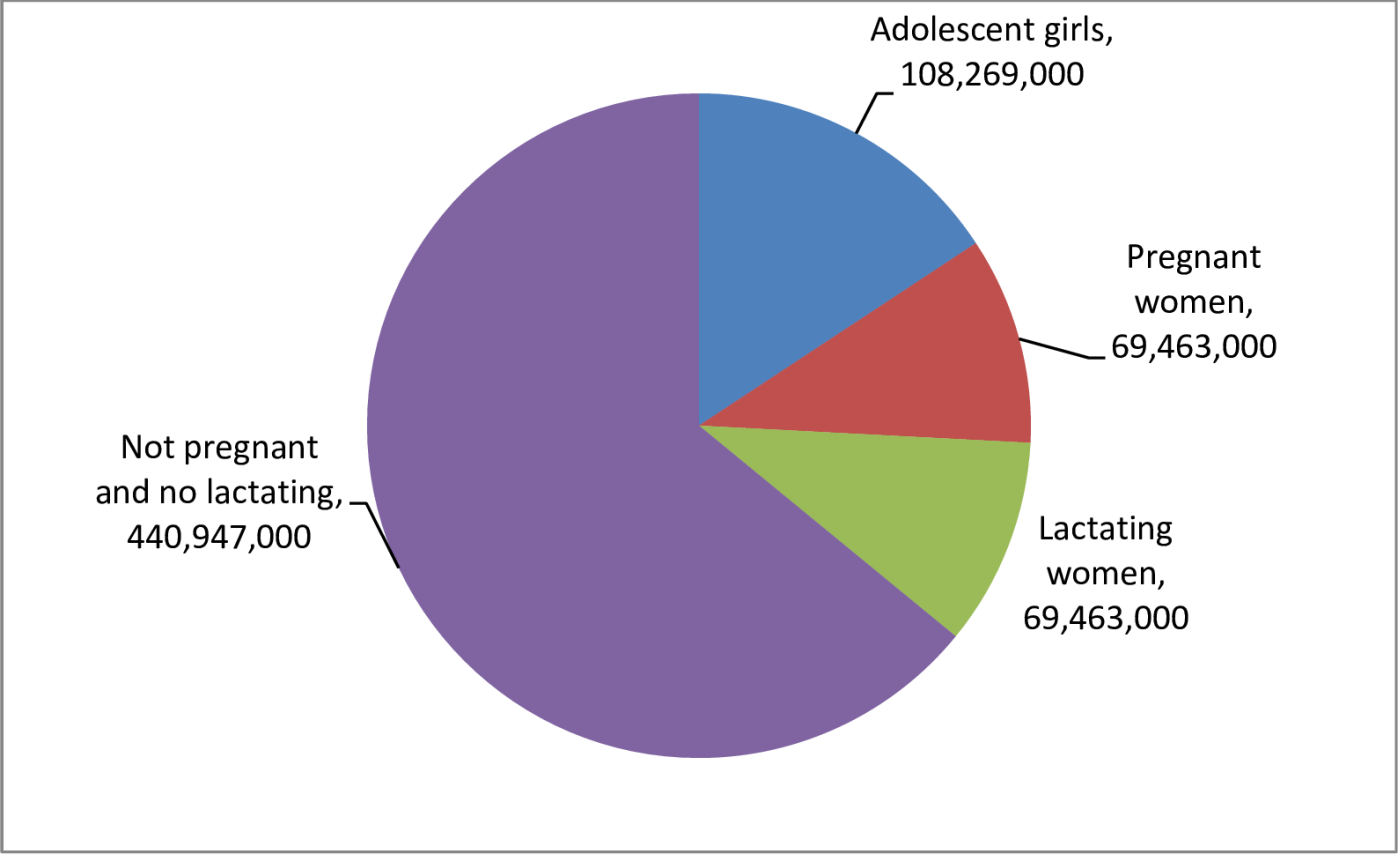
Estimated number of women of reproductive age requiring preventive chemotherapy for STH, by WRA categories, globally, in 2015.

**Fig 4:**
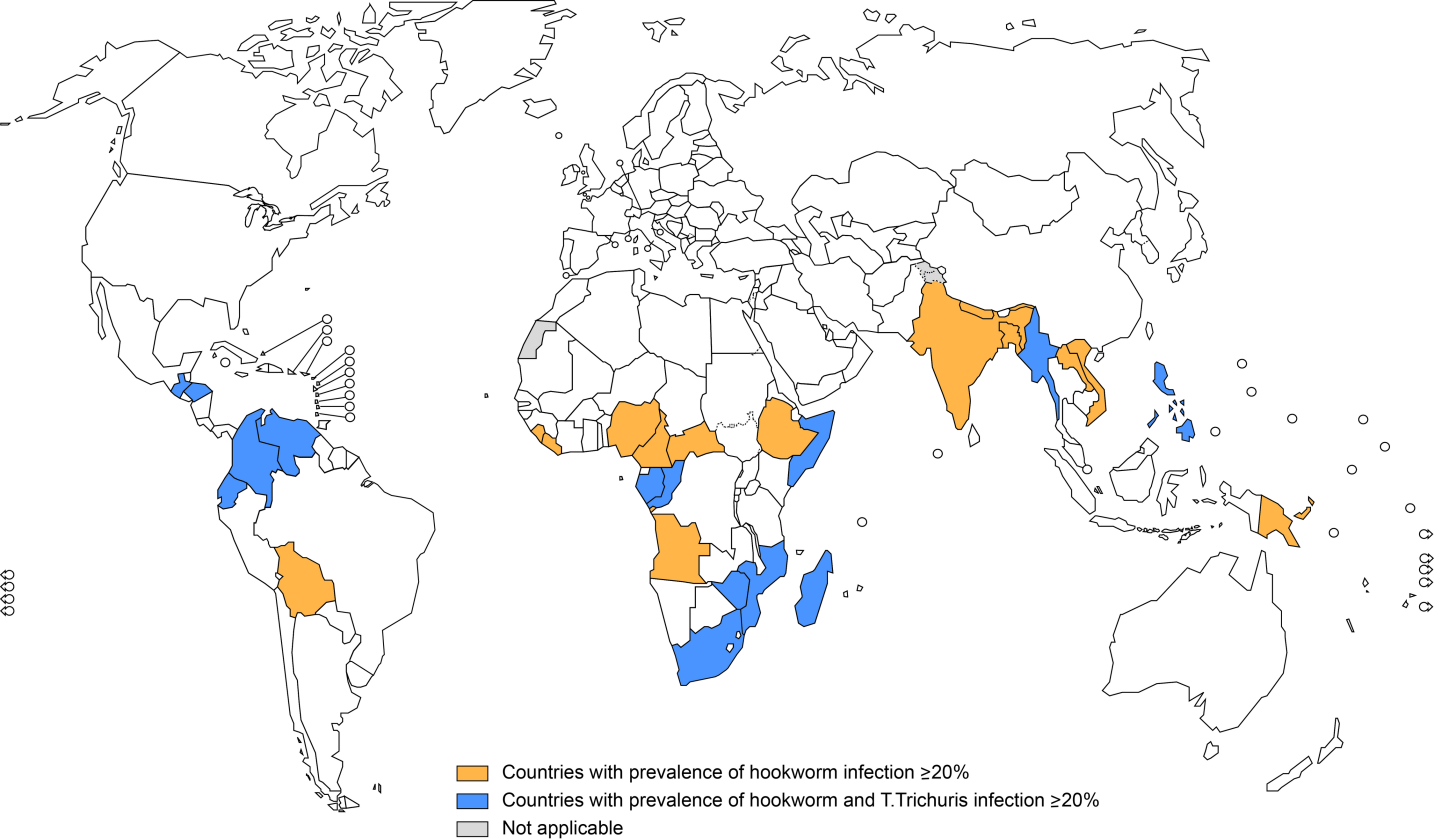
World map showing areas where hookworm and *T*. *trichiura* infections have a prevalence of over 20%, data from *Pullan et al* [6].

Globally, it is estimated that 688 million WRA in 102 countries were in need of PC for STH in 2015. This corresponds to approximately half of the total population of WRA in these countries (Fig 2). The South-East Asia (49%) and Africa regions (26%) have the largest number. Among the 688 million WRA in need of PC for STH, 10% are estimated to be pregnant, while 10% are lactating. Adolescent girls are estimated to represent 16%, while adult WRA who are neither pregnant nor lactating, are estimated to be 64% (Fig 3). Further, the estimated number of pregnant women in areas where the prevalence of hookworm infection and *T*. *trichiura* is equal to or greater than 20%, is estimated to be 26.7 million (Table 1, Fig 4).

**Table 1:** Estimated number of pregnant women in need of preventive chemotherapy for STH in areas where hookworm and *T*. *trichiura* prevalence is equal to, or greater than, 20%, 2015.

WHO Region	Number of STH-endemic countries	Estimated number of pregnant women in need of PC for STH
**Africa**	42	15,384,000
**Americas**	25	544,000
**Eastern Mediterranean**	7	473,000
**Europe**	5	0
**South-East Asia**	8	7,433,000
**Western Pacific**	15	2,919,000
**Total**	102	26,753,000

### Number of WRA treated for STH through the GPELF

Using data provided by the GPELF [15], it was estimated that, in 2015, more than 140 million WRA were dewormed with albendazole. The highest coverage rates were observed mostly in South-East Asian (27.5%) and African (24.8%) countries (Table 2).

**Table 2:** Coverage of preventive chemotherapy for STH through the LF programme in women of reproductive age, by WHO region, 2015.

WHO region	Number of WRA in need of PC for STH	Number of WRA treated for STH through the LF programme	Treatment coverage (%)
**Africa**	175,844,000	43,523,000	24.8
**Americas**	47,310,000	1,443,000	3.1
**Eastern Mediterranean**	42,186,000	0	0.0
**Europe**	3,052,000	NA	NA
**South-East Asia**	338,354,000	93,016,000	27.5
**Western Pacific**	81,396,000	3,017,000	3.7
**Total**	688,142,000	140,999,000	**20.5**

NA = not applicable

### Africa Region (AFR)

STH infection is endemic in 42 countries in Africa and the number of WRA in need of PC for STH is estimated to be 176 million (80% of the total WRA population in Africa) (Table 3). The number of pregnant women in areas where hookworm and *T*. *trichiura* prevalence is 20% or more is estimated at 10.2 million (Table 1) which represents more than 50% of the total number of pregnant women in need of PC for STH in the region.

**Table 3:** Estimated total number of women of reproductive age (WRA) in need of preventive chemotherapy (PC) for STH, by WRA sub-group and WHO region,2015.

	WRA in need of PC for STH, by WRA sub-group and WHO region, 2015				
WHO region	Adolescent girls	Pregnant women	Lactating women	Adult women not pregnant not lactating	Total
**Africa**	30,924,000	27,999,000	27,999,000	88,922,000	175,844,000
**Americas**	6,893,000	3,116,000	3,116,000	34,186,000	47,311,000
**Eastern Mediterranean**	8,778,000	6,617,000	6,617,000	20,174,000	42,186,000
**Europe**	360,000	249,000	249,000	2,193,000	3,051,000
**South- East Asia**	50,816,000	26,066,000	26,066,000	235,406,000	338,354,000
**Western Pacific**	10,498,000	5,416,000	5,416,000	60,066,000	81,396,000
**Total**	108,269,000	69,463,000	69,463,000	440,947,000	688,142,000

In 2015, 43.5 million WRA received albendazole through the GPELF which represents 24.8% of all WRA in need of PC for STH (Table 2).

### Americas Region (AMR)

STH infection is endemic in 25 countries in the Americas region. Approximately 47 million (31%) WRA require PC for STH. Among the three million pregnant women in need of treatment, approximately 540,000 are estimated to live in areas where hookworm and *T*. *trichiura* prevalence equals or exceeds 20% (Table 1).

LF is endemic in only 4 countries in the region and only limited areas within these countries require MDA. In 2015, in this region, 1.5 million WRA were treated through the GPELF. This represents about 3% of the total WRA population at risk of STH infection (Table 4).

**Table 4:** Estimated number of women of reproductive age in need of preventive chemotherapy who had been treated through GPELF, by WHO region, 2015.

	WRA in need of PC for STH treated through GPELF, by WRA sub-group and WHO region, 2015				
WHO region	Adolescents girls	Pregnant women	Lactating women	Adult women not pregnant not lactating	Total
**Africa**	10,910,000	NA	7,880,000	24,734,000	43,523,00
**Americas**	245,000	NA	94,000	1,104,000	1,443,000
**Eastern Mediterranean**	nr	NA	nr	nr	nr
**Europe**	NA	NA	NA	NA	NA
**South- East Asia**	16,844,000	NA	7,608,000	68,564,000	93,016,000
**Western Pacific**	344,000	NA	162,000	2,511,000	3,017,000
**Total**	28,343,000	NA	15,744,000	96,913,000	141,000,000

### Eastern Mediterranean Region (EMR)

Seven countries are endemic for STH infection in this region and 42 million WRA (56%) are in need of PC for STH (Table 3). For pregnant women in need of PC for STH, only approximately 470,000 are estimated to live in areas where hookworm and *T*. *trichiura* prevalence equals or exceeds 20% (Table 1).

In 2015, no data on treatment in WRA were reported to WHO.

### Europe Region (EUR)

Three million WRA (20%) in five endemic countries have been determined to be in need of deworming for STH (Table 3). In this region, neither LF nor hookworm infections are endemic in any country.

### South-East Asia Region (SEAR)

In this region, eight countries are endemic for STH and 338 million WRA (70%) are in need of PC for STH. Among the 26 million pregnant women in need of PC for STH, only 7.4 million are estimated to live in areas where hookworm and *T*. *trichiura* prevalence is 20% or more (Table 1).

In 2015, 27.5% WRA in need of PC for STH were dewormed through the GPELF (Table 4).

### Western Pacific region (WPR)

The Western-Pacific Region has 15 countries that are endemic for STH with an estimated 81 million WRA (19%) in need of PC for STH (Table 3).

In 2015, only 4% of WRA were dewormed through the GPELF (Table 4).

## Discussion

In 1996, The World Health Organization recommended that deworming be provided to pregnant women (after the first trimester) in areas where the prevalence of hookworm and *T*. *trichiura* infection exceeds 20–30% [19]. This recommendation has been re-emphasized in the new WHO guidelines on preventive chemotherapy to control STH in high-risk groups [20]. In 2001, the World Health Assembly delegates unanimously endorsed a resolution (WHA54.19) urging endemic countries to address the disease burden caused by soil-transmitted helminth infections in children and women of reproductive age [23]. Preventive chemotherapy targeting WRA is not yet being implemented in many STH-endemic countries despite the recognized benefits of deworming [24]. A recent simulation study, based on WORMSIM models, concluded that the control of hookworm infection by 2020 is possible if the current PC strategy also includes WRA [25].

Women of reproductive age are a large and diverse group of individuals who are at different stages in their reproductive life. Each stage presents different challenges for PC programmes in terms of delivery strategies [13]. For this reason, in this paper, we have differentiated WRA into four sub-groups (adolescent girls, pregnant women, lactating women and adult not pregnant and not lactating women), recognizing that each group requires a specific approach in terms of programme implementation: Adolescents girls could be reached through school-based deworming programmes by extending the targeted age groups to include the 15-to-19 year-old age group and/or integrating this age group into existing community-based programmes. India is one of the countries which has developed a community-based strategy addressing anaemia among WRA by distributing deworming drugs twice a year [26]. Pregnant and lactating women are perhaps the most easily reachable sub-groups since they are in contact with the health care system through antenatal and postpartum care services [27, 28]. In the new antenatal care guidelines, WHO recommends deworming of pregnant women after the first trimester in those areas where STH prevalence is greater than 20% [28]. To date, few countries (e.g. Cambodia, India, Sri Lanka, Vietnam) [29, 30] have put in place a national policy for deworming WRA through antenatal care programmes. However, details on these policies and on how the deworming programmes are implemented and monitored are not well documented for external public health assessments or uptake. Non-pregnant and non-lactating women are sub-groups not easily reachable through the health care system but community- based programmes such as the mass drug administration of the GPELF have achieved some success in this regard. During the LF MDA, pregnant women and women in the first week of breastfeeding after delivery are initially excluded but become eligible for treatment subsequently. Deworming through the LF MDA approach, however, is expected to change rapidly over the next few years as more districts implement LF transmission assessment surveys and stop treatments. The estimated contribution of 140 million WRA being treated through LF MDA will therefore decline. Affected countries will need to plan now on how their risk groups will continue to be reached after achieving LF elimination targets. Meanwhile, countries that are not LF-endemic should identify other platforms for deworming drug distribution in order to reach WRA which are adapted to the national context.

### Limitations

The WHO age interval for this group (15–49 years), is large and fertility is lower toward the end of the interval; for this reason probably the women in this part of the interval are less in need of deworming. We recognise that not all WRA estimated at risk of STH infection are infected. Only a proportion are infected and less will harbour moderate and heavy infection. However, this is one of the principles of preventive chemotherapy: ensuring the population at risk has access to treatment maximising the coverage (and benefit) of the infected population in the most cost-effective public health approach [2].

The assumption that, in an STH- endemic country, the proportion of WRA in need of treatment is the same as the proportion of school-age children in need of treatment is due to the paucity of epidemiological data specifically collected in women. While this assumption may introduce over- or under- estimates, on balance and in light of few WRA-specific data, we think this is a reasonable assumption to make. We hope that future studies will be able to confirm this assumption.

The data used to identify countries with a high prevalence of hookworm and *T*. *trichiura* infection were not all derived from nationally representative surveys. This could result in an over- or under-estimation of people affected by these infections. As countries strengthen their capacity to implement epidemiological surveys, we anticipate that these estimates will improve.

Lastly, the assumption that all pregnant women breastfeed for one year after delivery could underestimate the total number of lactating women since some of them will continue after one year, we could not find data on breastfeeding rate by country. We hope that, as countries start their deworming programme for WRA, data will be available and will be updated.

Despite these limitations, we think that having an estimation of the numbers of women in the four distinct sub-groups of WRA represents an important starting point which will guide discussions on appropriate strategies, facilitate planning and allocation of resources to control STH in WRA.
